# Mesenchymal Stromal Cells and Their Extracellular Vesicles Enhance the Anti-Inflammatory Phenotype of Regulatory Macrophages by Downregulating the Production of Interleukin (IL)-23 and IL-22

**DOI:** 10.3389/fimmu.2018.00771

**Published:** 2018-04-12

**Authors:** Kati Hyvärinen, Minna Holopainen, Vita Skirdenko, Hanna Ruhanen, Petri Lehenkari, Matti Korhonen, Reijo Käkelä, Saara Laitinen, Erja Kerkelä

**Affiliations:** ^1^Finnish Red Cross Blood Service, Helsinki, Finland; ^2^Molecular and Integrative Biosciences Research Programme, Faculty of Biological and Environmental Sciences, University of Helsinki, Helsinki, Finland; ^3^Minerva Foundation Institute for Medical Research, Biomedicum, Helsinki, Finland; ^4^Institute of Clinical Medicine, Division of Surgery, University of Oulu, Oulu, Finland; ^5^Department of Anatomy and Cell Biology, Institute of Biomedicine, University of Oulu, Oulu, Finland; ^6^Clinical Research Center, Department of Surgery and Intensive Care, Oulu University Hospital, Oulu, Finland

**Keywords:** regulatory macrophages, mesenchymal stromal cells, extracellular vesicles, interleukin-23, prostaglandin E_2_, resolution

## Abstract

Resolution-phase macrophage population orchestrates active dampening of the inflammation by secreting anti-inflammatory and proresolving products including interleukin (IL)-10 and lipid mediators (LMs). We investigated the effects of both human bone marrow-derived mesenchymal stromal cells (MSCs) and MSC-derived extracellular vesicles (MSC-EVs) on mature human regulatory macrophages (Mregs). The cytokines and LMs were determined from cell culture media of Mregs cultivated with MSCs and MSC-EVs. In addition, the alterations in the expression of cell surface markers and the phagocytic ability of Mregs were investigated. Our novel findings indicate that both MSC coculture and MSC-EVs downregulated the production of IL-23 and IL-22 enhancing the anti-inflammatory phenotype of Mregs and amplifying proresolving properties. The levels of prostaglandin E_2_ (PGE_2_) were substantially upregulated in MSC coculture media, which may endorse proresolving LM class switching. In addition, our results manifest, for the first time, that MSC-EVs mediate the Mreg phenotype change *via* PGE_2_. These data suggest that both human MSC and MSC-EVs may potentiate tolerance-promoting proresolving phenotype of human Mregs.

## Introduction

Inflammation is a crucial component of host tissue response, and controlling its initiation, progress, resolution, and post-resolution phases is essential for recovering tissue homeostasis. The overlapping stages of the cascade are moderated by macrophages, which are highly versatile and dynamic cells responding to various microenvironmental stimuli (1, 2). Macrophages exist as a heterogeneous population and display a spectrum of phenotypes both *in vivo* and *in vitro* depending on the provided signals. Conventional terms for two paradigmatic populations include classically activated “host defense” M1 and alternatively activated “wound-healing” M2. Additional concepts of “regulatory macrophages” or “Mregs” have emerged within the last decade (3–6).

At the resolution phase, the macrophage population shifts toward a resolving phenotype (7). These immune regulatory macrophages (Mregs) are characterized by immunosuppressive properties, such as high production of interleukin (IL)-10 and transforming growth factor (TGF)-β, and a downregulated production of pro-inflammatory IL-12 (3, 8, 9). The induction of Mreg populations may follow both innate and adaptive immune responses and arise from various stimuli including glucocorticoids, immune complexes, prostaglandins (PGs), IL-10, and apoptotic cells, combined with a second stimulus, such as a toll-like receptor ligand (3, 9–12). In recent years, Hutchinson and coworkers have established an experimental method for the preparation of *ex vivo*-manipulated regulatory macrophages. These cells suppress mitogen-stimulated T-cell proliferation *in vitro* and have been used as a promising immunosuppressive agent in early-phase clinical trials in renal transplantations (6, 13).

In addition to anti-inflammatory cytokines, lipid mediators (LMs) play an important role in the resolution phase. The resolution is initiated with LM class switching, in which PGs act as a cue for the conversion of pro-inflammatory to proresolving LM production. PGE_2_ and PGD_2_ induce neutrophils to produce fewer pro-inflammatory 5-lipoxygenase (5-LOX)-derived LMs, such as leukotrienes, and increase the production of 15-LOX products, such as lipoxins (LXs), through cyclic adenosine monophosphate induction and regulation of the gene transcription of 15-LOX (14). Proresolving LMs, termed specialized proresolving mediators (SPMs), reduce inflammation by decreasing neutrophil recruitment and increasing macrophage-mediated phagocytosis and efferocytosis (15). Macrophages are known to produce SPMs such as LXs, resolvins (Rvs), protectins, and maresins (16).

Mesenchymal stromal cells (MSCs) are multipotent adult stem cells that have been widely used in experimental cell therapy due to their immunosuppressive and anti-inflammatory properties (17). Key players in MSC immunomodulation include the tryptophan-degrading enzyme indoleamine 2,3-dioxygenase, adenosine-producing CD73, and PGE_2_ (18–22). MSCs are able to polarize macrophages toward a more anti-inflammatory phenotype in a PGE_2_-mediated manner (23–25). MSCs may improve the phagocytosis of macrophages by transporting mitochondria to macrophages *via* tunneling nanotube-like structures (26). MSCs have also been reported to produce SPMs in a murine model (27), but the evidence on SPM biosynthesis in human MSCs is limited, and only the production of an important proresolving mediator LXA_4_ has been described (28). In addition to secreted soluble molecules, paracrine activity *via* extracellular vesicles (EVs) is an important function of MSCs. MSC-derived EVs (MSC-EVs) mediate the immunosuppressive effect of MSCs (29, 30) and may also elicit a similar therapeutic response as the cells themselves (31–33). Lo Sicco et al. recently reported that human MSC-EVs are able to trigger polarization from the M1 to M2 phenotype in a murine model both *in vitro* and *in vivo* (34).

Mregs are considered an important proresolving cell population during the later stages of the immune response. Despite this prominent role, the cooperation between Mregs and other well-known immunomodulatory agents, such as MSCs, is sparsely studied. The majority of previous research on the effects of MSCs has been executed in murine models or by observing M2-type switch using polarized monocytes. Especially, the effect of MSCs or MSC-EVs on the properties of mature Mregs has not been addressed before. In this study, we focused on interplay in resolution and investigated the effects of human MSC coculture and MSC-EVs on the human Mreg population. The levels of cytokines and LMs were analyzed from conditioned media. In addition, we evaluated phagocytic ability and the alterations of phenotype marker expression of the Mreg population. Our novel findings indicate that both MSC coculture and MSC-EVs enhance the anti-inflammatory phenotype of Mregs by downregulating the production of IL-23 and IL-22. We identified several LMs and pathway markers from human Mreg-, MSC-, and EV-conditioned media. The results manifest that MSC-EVs may also mediate the discovered changes in cytokine levels *via* PGE_2_, and thus promote the resolution of inflammation.

## Materials and Methods

### MSC Culture

Human bone marrow-derived MSCs from two donors (35, 36) at passage 4 were thawed, and 1,200 cells/cm^2^ were plated on 10 cm plates (Nunclon™ Delta Surface, Thermo Fisher Scientific) in 10 ml α-MEM (Gibco) supplemented with 10% fetal bovine serum (FBS) (Gibco), 20 mM HEPES (Gibco), 100 U/ml penicillin, and 100 µg/ml streptomycin (Gibco). The cells were incubated at 37°C, 5% CO_2_ for 5 days, and the medium was renewed after 24 h. The cells were washed with 5 ml warm endotoxin-free phosphate-buffered saline (PBS) with or without (w/o) Ca^2+^/Mg^2+^ (Gibco DPBS CTS™) and detached with 1.5 ml TrypLE™ Express (Gibco). The detachment process was stopped with 5 ml warm 10% FBS (Sigma-Aldrich) in RPMI Medium 1640, GlutaMAX™ Supplement (Gibco), and the cells were centrifuged at 500 *g* for 5 min. The pellet was suspended with 10% FBS in RPMI Medium 1640, GlutaMAX™ Supplement, and cells were counted with NucleoCounter^®^ NC-100™ (ChemoMetec).

### MSC-Derived Extracellular Vesicle Extraction

Human bone marrow-derived MSC from two donors (35, 36) at passage 4 were thawed, and 1,200 cells/cm^2^ were plated on two 15 cm plates in 30 ml α-MEM supplemented with 10% FBS, 20 mM HEPES, 100 U/ml penicillin, and 100 µg/ml streptomycin. The cells were incubated at 37°C, 5% CO_2_ for 6 days, and the medium was renewed after 24 h. The cells were detached with TrypLE™ Express, and 1,300 cells/cm^2^ were plated on two-chamber type of Corning^®^ CellSTACK^®^ cell culture chambers (Sigma-Aldrich) in 250 ml medium. The chamber cultivation continued at 37°C, 5% CO_2_ for 3 days, and the medium was renewed after 24 h. Before starvation, the cells were washed three times with 100 ml PBS and once with 75 ml α-MEM. During starvation, the cells were incubated in 200 ml serum-free starvation medium α-MEM at 37°C, 5% CO_2_ for 2 days. The media were collected and centrifuged at 2,000 *g* for 10 min to remove cell debris. The supernatant was ultracentrifuged with Optima™ MAX-XP Ultracentrifuge (Beckman Coulter) at 100,000 *g* 1.5 h +4°C with MLA-50 rotor (*k*-factor = 92, Beckman Coulter), and the pelleted EVs were combined. For the second EV collection, the cell starvation was continued in 200 ml α-MEM at 37°C, 5% CO_2_ for 2 days followed by replication of EV centrifugation steps.

### Macrophage Polarization Assay

The schematic overview of macrophage polarization assay is presented in Figure S1 in Supplementary Material, and the detailed method is described in Supplementary Methods in Supplementary Material.

Briefly, human peripheral blood mononuclear cells (PBMC) were extracted from buffy coats using Ficoll-Pague™ Plus (GE Healthcare Life Sciences) density gradient centrifugation at day 0. Monocyte selection was performed by plating 2 × 10^6^ PBMC/well in Nunclon™ Delta Surface 24-well plates (Thermo Fisher Scientific) in RPMI Medium 1640 (Gibco), incubating at 37°C, 5% CO_2_ for 2 h. The attached monocytes were incubated at 37°C, 5% CO_2_ for 6 days in the following Polarization Media: 5 ng/ml M-colony stimulating factor (CSF) (PromoCell), 10% FBS (Sigma-Aldrich) in RPMI Medium 1640, GlutaMAX™ Supplement for Mreg polarization; 50 ng/ml GM-CSF (PromoCell), 10% FBS in RPMI Medium 1640, GlutaMAX™ Supplement for M1 polarization; and 50 ng/ml M-CSF, 10% FBS in RPMI Medium 1640, GlutaMAX™ Supplement for M2 polarization.

At day 6, the media were replaced with the following Activation Media: 25 ng/ml interferon (IFN)-γ, 10 ng/ml lipopolysaccharide (LPS), 5 ng/ml M-CSF, 10% FBS in RPMI Medium 1640, GlutaMAX™ Supplement for Mreg polarization; 50 ng/ml IFN-γ, 10 ng/ml LPS, 50 ng/ml GM-CSF, 10% FBS in RPMI Medium 1640, GlutaMAX™ Supplement for M1 polarization; and 20 ng/ml IL-4, 50 ng/ml M-CSF, 10% FBS in RPMI Medium 1640, GlutaMAX™ Supplement for M2 polarization. The added media volume was 500 µl in treatment wells and 600 µl in control wells. The incubation was continued at 37°C, 5% CO_2_.

At day 7, 20,000 MSCs in 100 µl Mreg/M1/M2 Activation media were added into representative treatment wells. The final media volume was 600 μl/well, and the cells were incubated at 37°C, 5% CO_2_ for 3 days. Alternatively, in MSC-EV supplementation experiments, at day 7, isolated EVs from one two-chamber were suspended in Mreg Activation Media, and 50 µl was added into 30 representative treatment wells. The supplementation was repeated at day 9, and isolated EVs in 50 µl representative Mreg Activation Media were added into each well. The final media volume was 600 μl/well, and the cells were incubated at 37°C, 5% CO_2_ for 3 days.

At day 10, media samples were collected from each well and centrifuged at 300 *g* for 15 min at RT. The supernatants were snap frozen on dry ice and stored at −70°C. Cell samples for flow cytometry analysis were detached with 0.5 ml/well cold Macrophage Detachment Solution DFX (PromoCell).

### Phagocytosis Assay

The phagocytic ability of Mregs was determined at day 10 of Macrophage assay using a Phagocytosis Assay Kit (IgG FITC) (Cayman Chemicals) according to the manufacturer’s instructions. Briefly, latex beads coated with FITC-labeled rabbit IgG were added in 1:300 dilution in 200 µl Polarization media II on Mregs and incubated at +37°C, 5% CO_2_ for 4 h in Cell-IQ^®^ automated cell culture and analysis system (CM Technologies Oy). After incubation, the cells were washed with 1 ml warm PBS and detached with 0.5 ml/well cold Macrophage Detachment Solution DFX. The cells were suspended up to 15 ml PBS and centrifuged at 350 *g* for 10 min. To quench cell-bound external fluorescence, the pellets were suspended with 45 µl Phagocytosis Kit Assay Buffer (Cayman Chemicals) and 5 µl 10× Trypan Blue Quenching Solution (Cayman Chemicals) was added followed by 1–2 min incubation at RT. The cells were washed with 2 ml Phagocytosis Kit Assay Buffer, centrifuged at 350 *g* for 10 min, and suspended in 50 µl 0.3% bovine serum albumin, 2 mM EDTA in PBS, pH 7.2.

### Macrophage Phenotyping Using Flow Cytometry Analysis

The antibody staining was performed with PE-CD80 (clone 2D10.4, mouse IgG1 k, eBioscience), PE-Cy7-CD86 [clone 2331 (FUN-1), mouse IgG1 k, BD Biosciences], BV421-CD163 (clone GHI/61, mouse IgG1 k, BD Biosciences), and APC-CD206 (clone 19.2, mouse IgG1 k, BD Biosciences) according to the manufacturers’ instructions. Respectively, conjugated isotype control antibodies were used as negative control for background staining. Macrophages were suspended in 50 µl staining buffer (0.3% bovine serum albumin, 2 mM EDTA in PBS, pH 7.2) and incubated with 2.5 µg of Human BD Fc Block™ (BD Biosciences) for 10 min at RT. The pre-mixed fluorescent antibody cocktail was added, and the cells were incubated on ice in darkness for 30 min. After staining, the cells were washed with 2 ml staining buffer, pelleted by centrifuging at 350 g for 10 min, and suspended in 100 µl staining buffer.

Cell data were acquired with BD FACSAria IIU (BD Biosciences) flow cytometer using FACSDiva™ version 8.0.1 software (BD Biosciences) and analyzed with FlowJo^®^ version 10.0.7 software (FlowJo, LLC). Macrophages were gated based on forward (FSC) and side (SSC) scatter patterns. Doublets and aggregates were excluded using FSC area versus FSC height. The fluorescence positive cells were gated based on isotype controls and populations. The results are represented as median fluorescence intensity (FRI) and frequency of positive cells. The representative gating strategy is presented in Figure S2 in Supplementary Material.

### Cytokine Measurements

The cell culture media from Macrophage polarization assay was analyzed for 18 cytokines using human Th1/Th2/Th9/Th17/Th22/Treg 18 Plex ProcartaPlex Immunoassay (eBioscience) according to the manufacturer’s instructions. The media samples were thawed on ice, and three technical replicates from each experiment were pooled together. Briefly, 50 µl of samples and standards was incubated with magnetic beads followed by washing steps and the detection antibody mixture. After addition of streptavidin-PE, the signal data were acquired on Luminex^®^ 100 system (Luminex). The data were analyzed with ProcartaPlex Analyst 1.0 Software (Thermo Fisher Scientific). Measures within the cytokine-specific detection range were included in the analysis, except the tumor necrosis factor (TNF)-α analysis, in which, for certain M1 samples, the upper limit of the detection range was used.

### Identification of LMs and Pathway Markers

The levels of LMs and pathway markers were determined using liquid chromatography–tandem mass spectrometry (LC–MS/MS). The media samples were analyzed for LMs thromboxane (Tx)B_2_, PGE_2_, PGD_2_, 15-deoxy-Δ^12,14^-PGJ_2_, leukotriene B_4_, LXA_4_, RvD1, RvD2, RvD3, maresin 1, 10*S*,17*S*-dihydroxydocosahexaenoic acid (diHDHA) (also known as protectin DX), and monohydroxy pathway markers 15-hydroxyeicosatetraenoic acid (HETE), 18-hydroxyeicosapentaenoic acid (HEPE), 17-HDHA, and 14*S*-HDHA.

The cell culture samples were thawed on ice, and three volumes of methanol containing 500 pg of internal standards d_4_-PGE_2_ and d_5_-RvD2 were added to the sample. Then, samples were incubated for 45 min at −20°C for protein precipitation. The samples were centrifuged at 700 *g* for 15 min, and the supernatant was filtered using Captiva ND Lipids filtration device (Agilent Technologies), which trapped phospholipids but allowed LMs to pass through. The eluate was then concentrated to 75 µl, and 5 µl was injected to the LC–MS/MS system. The LMs and pathway markers were analyzed by using 6490 Triple Quadrupole LC/MS equipment with Agilent Jet Stream and iFunnel Technology coupled with 1290 Infinity LC (Agilent Technologies). For chromatographic separation, a ZorBAX Eclipse Plus C18 RRHD analytical column (2.1 mm × 50 mm, 1.8 µm, Agilent Technologies) was used. The LC–MS/MS method employed multiple reaction monitoring (MRM) detection for each LM (Table S1 in Supplementary Material) with the LC phases and optimized source parameters described by Le Faouder et al. (37).

Samples were analyzed as triplicates. The criteria used to identify a peak were as follows: (1) retention time of the peak was matched with the standard, (2) a peak eluted at the correct retention time in both Quantifier and Qualifier MRM scans of a single mediator, and (3) all of the triplicate sample runs contained a peak. The data were analyzed with MassHunter Quantitative Analysis software version 6.00 (Agilent Technologies) using the Quantifier MRM for quantitative analysis (Q3 quant, Table 3). Concentrations were normalized against the respective internal standards (Table S1 in Supplementary Material), and the detection limit was 0.2 ng/ml.

### Statistical Analyses

Statistical analyses were performed with GraphPad PRISM^®^ version 7.02 (GraphPad Software). Due to a relatively low number of biological replicates and non-normal distribution of variables, non-parametric statistical methods were applied. The values of cell culture media cytokines were log10-transformed. The variation of cytokines and flow cytometry results between M1-, M2-, and Mreg-conditioned media groups was analyzed by the Kruskal–Wallis test, and pairwise analyses were executed using the Mann–Whitney *U* tests. When assessing the variation of the levels of cytokines and LMs in Mreg-conditioned media w/o MSC coculture or MSC-EVs, the Wilcoxon matched-pairs signed-rank test was used. The results are expressed as median with interquartile ranges. *p*-Values < 0.05 were considered significant.

### Ethical Permits

BM MSC donors gave their voluntary, informed, and written consent before sample collection, and the study protocols were approved by the Ethical Committee of Northern Ostrobothnia Hospital District or Ethical Committee of Hospital District of Helsinki and Uusimaa, Finland. The utilization of anonymized PBMCs from blood donors in research is in accordance with the rules of the Finnish Supervisory Authority for Welfare and Health (Valvira).

## Results

### Characteristics of Mregs and Other Macrophage Subtypes

**Figure 1:**
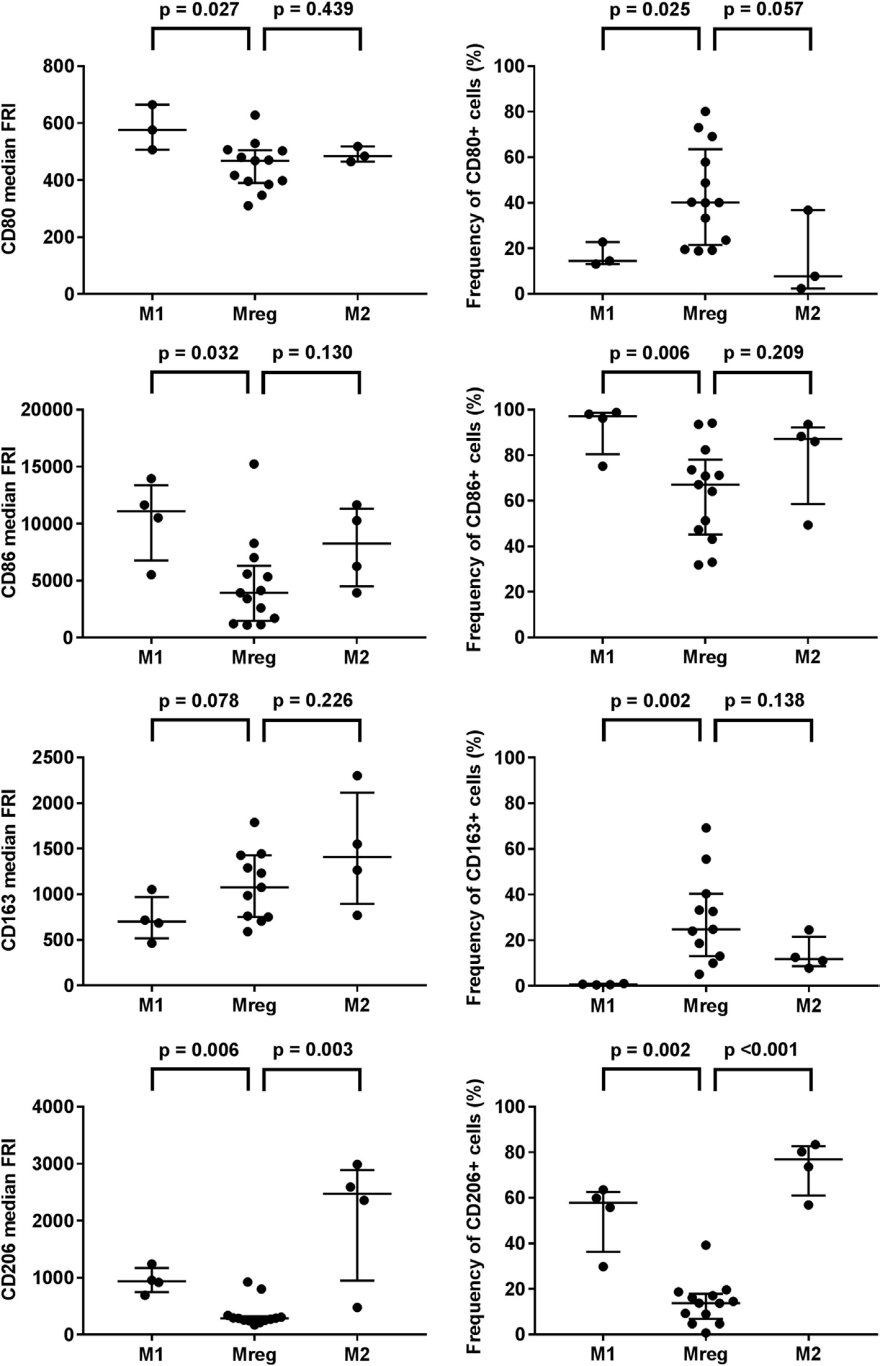
Phenotypes of M1, M2, and Mreg subtypes. The antibody staining was performed with PE-CD80, PE-Cy7-CD86, BV421-CD163, and APC-CD206 according to the manufacturers’ instructions. The median fluorescence intensities (left panel) and frequencies of positive cells (right panel) were determined with flow cytometry analysis. The significance in pairwise variation between M1 and Mreg, and M2 and Mreg was analyzed using the Mann–Whitney *U* test. The results are presented as median with interquartile range. The number of biological replicates varied from 3 to 13. Abbreviations: FRI, fluorescence intensity; M1, classically activated “host defense” macrophage; M2, alternatively activated “wound-healing” macrophage; Mreg, regulatory macrophage.

Phenotypes of Mreg, M1, and M2 were assessed by the flow cytometry analysis of T cell activation co-stimulatory molecules CD80 and CD86, scavenger receptor CD163, and mannose receptor CD206, and the results are presented in Table 1. The observed Mreg phenotype was CD80^low/intermediate^, CD86^+^, CD163^low^, and CD206^low^. Approximately 40% of Mregs were positive for CD80, 70% for CD86, 25% for CD163, and 14% for CD206 expression. All the median FRIs and the frequencies of positive cells significantly differed between the studied macrophage subtypes, except the median FRI of CD80 (Table 1). When analyzing the subtypes pairwise (Figure 1), we observed that the median FRI of CD80 was higher among M1 than among Mreg (*p* = 0.027). All macrophage subtypes were highly positive for CD86. Both the median FRI of CD86 (*p* = 0.032) and frequency of CD86 positive cells (*p* = 0.006) were higher among M1 than Mreg.

**Table 1:** Characteristics of Mregs and other macrophage subtypes.

Protein	M1 (*n* = 3–4)	M2 (*n* = 3–4)	Mreg (*n* = 13)	
	**Median FRI (IQR)**			***p*-Value^a^**
CD80	576 (158)	484 (53)	468 (196)	0.060
CD86	11,085 (6,604)	8,262 (6,803)	3,950 (4,845)	0.039
CD163	702 (451)	1,409 (1,220)	1,076 (679)	0.047
CD206	937 (423)	2,476 (1,941)	287 (74)	<0.001

	**Median frequency of positive cells (%)**			***p*-Value^a^**
CD80	15 (10)	8 (34)	40 (42)	0.011
CD86	97 (18)	87 (34)	67 (33)	0.012
CD163	1 (0)	12 (13)	25 (27)	<0.001
CD206	58 (26)	77 (22)	14 (11)	<0.001

*FRI, fluorescence intensity; IQR, interquartile range; M1, classically activated “host defense” macrophage; M2, alternatively activated “wound-healing” macrophage; Mreg, regulatory macrophage*.

*^a^The significance in variation between M1, M2, and Mreg was analyzed using the Kruskal–Wallis test*.

The observed phenotype differences were more prominent between Mreg and M1 than between Mreg and M2. The major exception was CD206; when analyzed pairwise, both median FRI (*p* = 0.003) and frequency of CD206^+^ cells (*p* < 0.001) were higher among M2 than Mreg.

**Figure 2:**
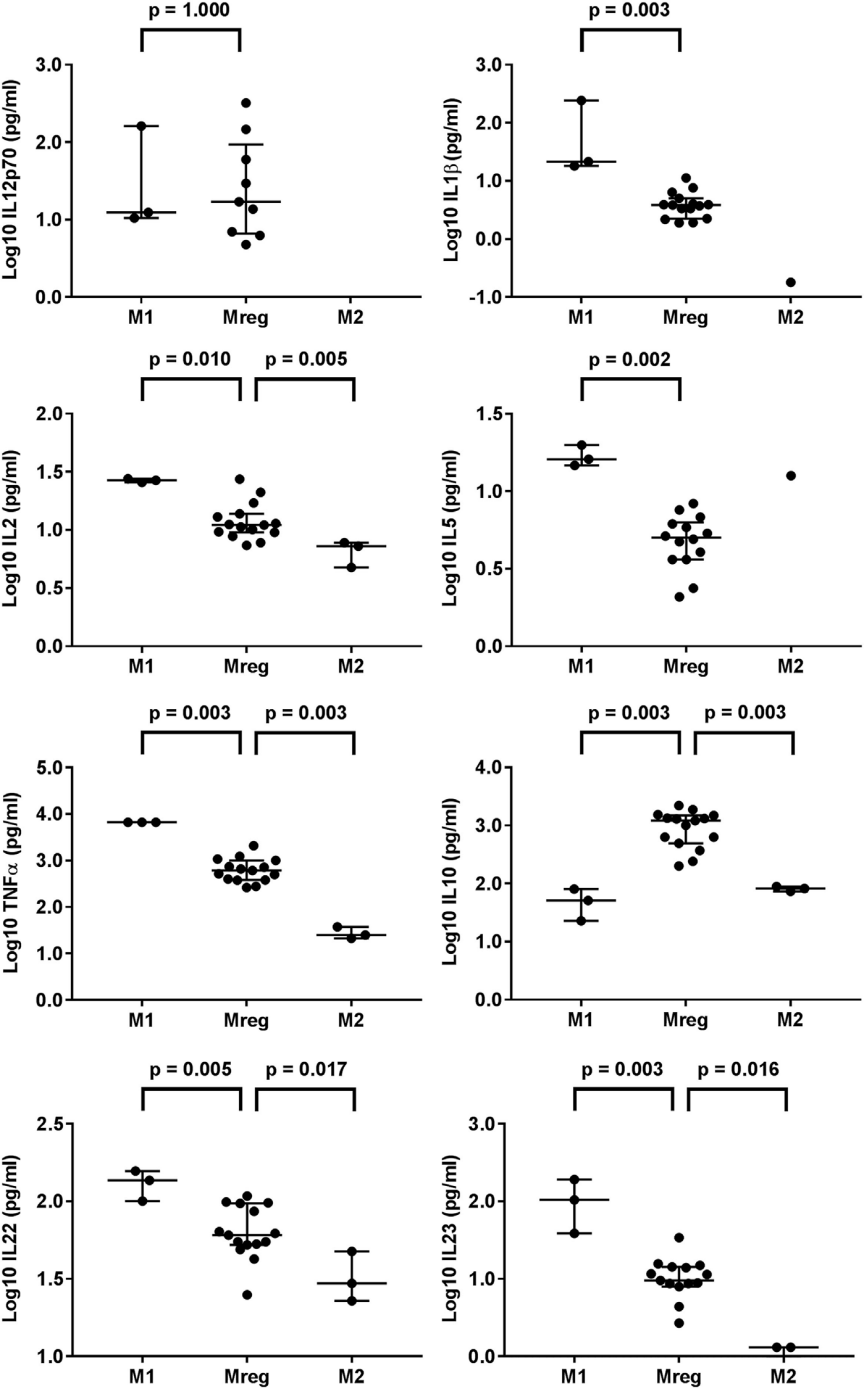
Level of cytokines in Mreg-, M1-, and M2-conditioned media. The media were analyzed for 18 cytokines, and measures within the cytokine-specific detection range were included in the analysis. The values of cell culture media cytokines were log10-transformed. The significance in pairwise variation between M1 and Mreg, and M2 and Mreg was analyzed using the Mann–Whitney *U* test, and the results are expressed as median with interquartile range. The number of biological replicates varies from 3 (for M1 and M2) to 15 for Mreg. Abbreviations: IL, interleukin; M1, classically activated “host defense” macrophage; M2, alternatively activated “wound-healing” macrophage; Mreg, regulatory macrophage.

The results of cytokines in Mreg-, M1-, and M2-conditioned media are presented in Figure 2. The levels of IL-1β, IL-2, IL-5, TNF-α, IL-10, IL-22, and IL-23 significantly varied between the macrophage-conditioned media groups. When compared pairwise, compared with M1-conditioned media, the levels of IL-1β (*p* = 0.003), IL-2 (*p* = 0.010), IL-5 (*p* = 0.002), TNF-α (*p* = 0.003), IL-22 (*p* = 0.005), and IL-23 (*p* = 0.003) in Mreg-conditioned media were significantly lower. By contrast, the level of IL-10 was higher in Mreg-conditioned media than in M1- and M2-conditioned media (*p* = 0.003 for both comparisons). In addition, the levels of IL-22 and IL-23 were higher in Mreg-conditioned media than in M2-conditioned media (*p* = 0.017 and *p* = 0.016, respectively).

### Effect of MSC Coculture or MSC-EVs on Mreg Phenotype

The effects of MSC coculture or MSC-EVs on Mreg CD80, CD86, CD163, and CD206 phenotype were assessed by flow cytometry analysis and the representative raw data overlays are presented in Figures S3 and S5 in Supplementary Material, respectively. The pairwise analyses are depicted in Table 2 and Figures S4 and S6 in Supplementary Material. The MSC coculture did not affect the median FRI of CD80 in Mregs; however, the frequency of CD80^+^ Mregs was lower in the coculture than in Mreg culture alone (*p* = 0.024). We observed no significant change in CD86 expression and frequency among Mregs. The median FRI of scavenger receptor CD163 expression significantly decreased in Mregs when cocultured with MSC (*p* = 0.003). In addition, the frequency of CD163^+^ Mregs was lower in the coculture than in Mreg culture alone (*p* = 0.002). The same trend was observed in mannose receptor CD206 expression and the frequency of positive Mregs, even though only the reduction in frequency was significant (*p* = 0.060 for CD206 median FRI; *p* = 0.008 for frequency of CD206^+^ Mregs).

**Table 2:** Effect of MSC coculture or MSC-EVs on Mreg phenotype.

Protein	Mregs w/o MSC coculture			Mregs w/o MSC-EVs		
	Mreg (*n* = 11)	Mreg + MSC (*n* = 11)		Mreg (*n* = 8)	Mreg + MSC-EV (*n* = 8)	
	**Median FRI (IQR)**		***p*-Value^a^**	**Median FRI (IQR)**		***p*-Value^b^**
CD80	417 (170)	443 (151)	0.365	524 (256)	548 (276)	0.188
CD86	3,950 (4,845)	2,998 (6,480)	0.414	2,601 (1,820)	3,593 (2,665)	0.109
CD163	1,076 (679)	730 (529)	0.003	1,012 (560)	967 (468)	0.016
CD206	287 (74)	245 (154)	0.060	264 (74)	245 (45)	0.031

	**Median frequency of positive cells (%)**		***p*-Value^a^**	**Median frequency of positive cells (%)**		***p*-Value^b^**
CD80	40 (36)	31 (44)	0.024	57 (30)	58 (36)	0.547
CD86	67 (33)	42 (39)	0.376	80 (21)	87 (13)	0.016
CD163	25 (27)	7 (12)	0.002	19 (15)	9 (12)	0.016
CD206	14 (11)	3 (3)	0.008	9 (8)	9 (2)	0.078

*EV, extracellular vesicle; IQR, interquartile range; Mreg, regulatory macrophage; MSC, mesenchymal stromal cell; FRI, fluorescence intensity; MSC-EVs, MSC-derived extracellular vesicles; w/o, with or without*.

*^a^The statistical significance of variation between Mregs and Mregs with MSC coculture was determined using the Wilcoxon matched-pairs signed-rank test*.

*^b^The statistical significance of variation between Mregs and Mregs with MSC-EVs was determined using the Wilcoxon matched-pairs signed-rank test*.

In concordance with the MSC coculture results, MSC-EVs decreased the median FRI of CD163 (*p* = 0.016), and compared with Mregs cultured without EVs, reduced the frequency of CD163^+^ Mregs (*p* = 0.016) (Table 2; Figure S6 in Supplementary Material). Again, the same trend was observed in CD206 expression and frequency of positive Mregs, even though only the reduction of median FRI of CD206 reached significance (*p* = 0.031 for CD206 median FRI; *p* = 0.078 for frequency of CD206^+^ Mregs).

### Effect of MSC Coculture and MSC-EVs on the Cytokine Levels of Mreg-Conditioned Media

**Figure 3:**
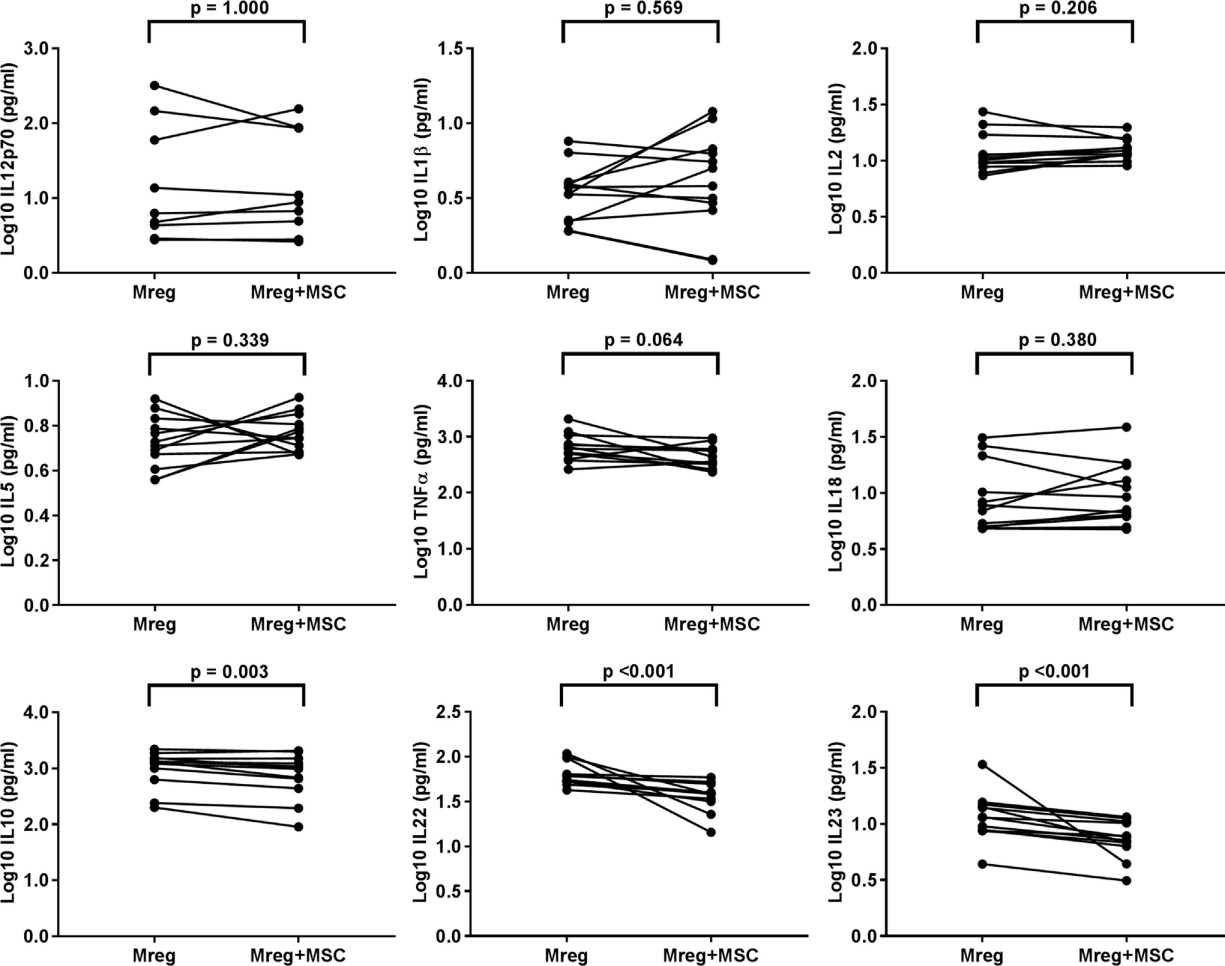
Effect of MSC coculture on the level of cytokines in Mreg-conditioned media. The media were analyzed for 18 cytokines and measures within the cytokine-specific detection range were included in the analysis. The values of cell culture media cytokines were log10-transformed. The variation of cytokines between Mreg-conditioned media with and without MSC coculture was analyzed by the Wilcoxon matched-pairs signed-rank test. The number of biological replicates varied from 9 to 12. Abbreviations: IL, interleukin; Mreg, regulatory macrophage; MSC, mesenchymal stromal cell.

The cytokine levels were measured from Mreg-conditioned media without MSC coculture, and the results are presented in Figure 3. The levels of IL-10, IL-22, and IL-23 were significantly lower in Mreg-conditioned media with MSC coculture than without it (*p* = 0.003, *p* < 0.001, and *p* < 0.001, respectively). In addition, there was a positive trend toward decreased level of TNF-α after MSC coculture, even though the result did not reach significance (*p* = 0.064). This effect was more prominent among M1 subtype where the TNF-α production was more abundant (data not shown).

**Figure 4:**
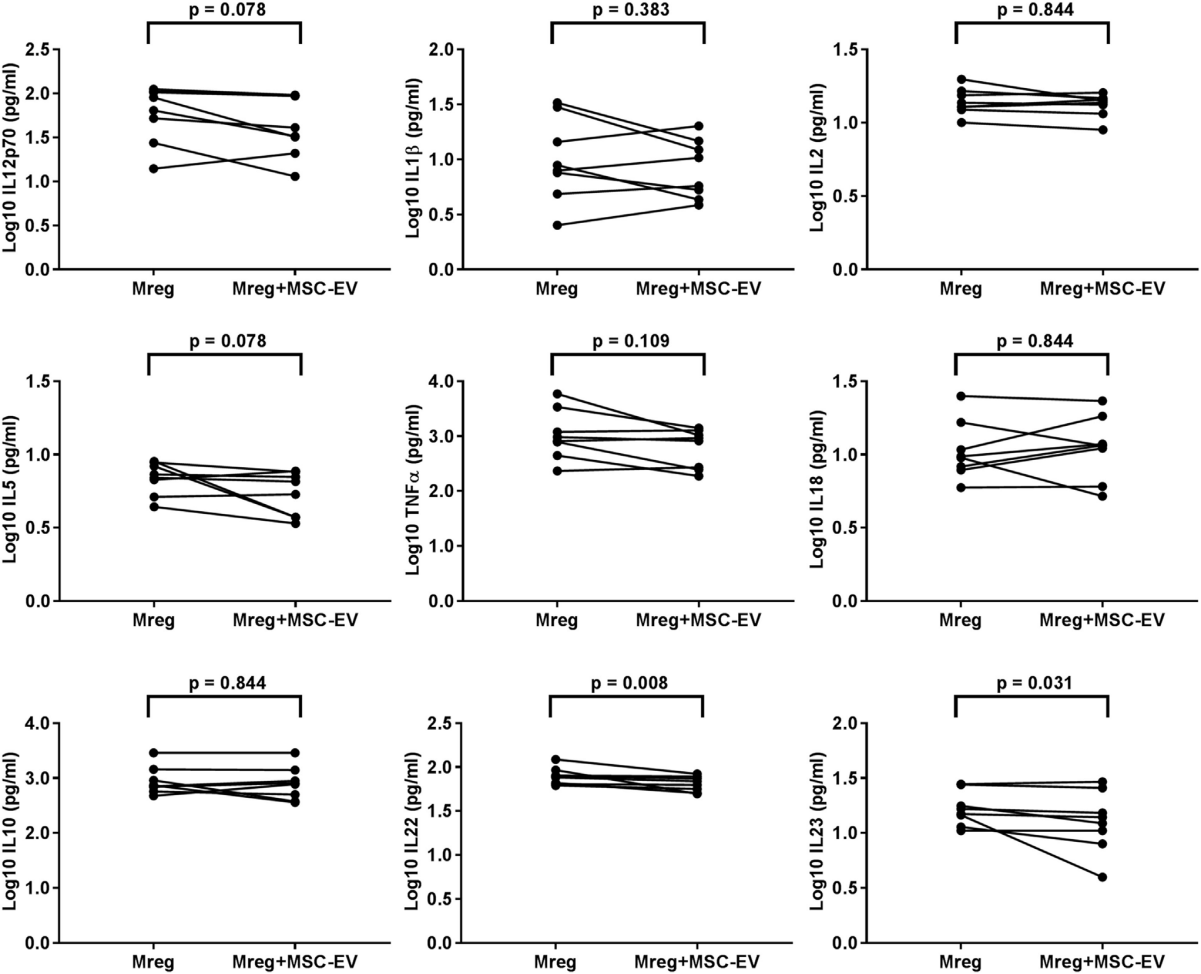
Effect of MSC-derived extracellular vesicles on the level of cytokines in Mreg-conditioned media. The media were analyzed for 18 cytokines, and measures within the cytokine-specific detection range were included in the analysis. The values of cell culture media cytokines were log10-transformed. The variation of cytokines between Mreg-conditioned media with and without MSC-EVs was analyzed by the Wilcoxon matched-pairs signed-rank test. The number of biological replicates is 8. Abbreviations: EV, extracellular vesicle; IL, interleukin; Mreg, regulatory macrophage; MSC, mesenchymal stromal cell; MSC-EVs, MSC-derived extracellular vesicles.

The cytokine levels were measured from Mreg-conditioned media w/o MSC-EVs and the results are presented in Figure 4. The levels of IL-22 and IL-23 were significantly lower in Mreg-conditioned media with MSC coculture than without it (*p* = 0.008 and *p* = 0.031, respectively).

### Effect of MSC Coculture and MSC-EVs on the LMs and Pathway Markers of Mreg-Conditioned Media

The levels of LMs and pathway markers were determined using LC–MS/MS, and identification of 7 out of the 15 metabolites addressed was successful. Table 3 displays the results for Mreg-conditioned media w/o MSC coculture or MSC-EVs. The levels of arachidonic acid-derived PGE_2_ (*p* = 0.002) and 15-HETE (*p* = 0.020), and docosahexaenoic acid-derived 17-HDHA (*p* = 0.009) were higher in Mreg-conditioned media with MSC coculture than in Mreg culture alone. When treated with MSC-EVs, compared with non-treated Mreg culture, the Mreg-conditioned media showed a significant increase in the level of PGE_2_ (*p* = 0.031).

**Table 3:** Levels of lipid mediators (LMs).

LM	Tandem mass spectrometry			Mreg-conditioned media w/o MSC coculture			Mreg-conditioned media w/o MSC-EVs		
	Mass/charge			Median (IQR) (ng/ml)^a^			Median (IQR) (ng/ml)^b^		
	Q1	Q3 (quant)	Q3 (qual)	Mreg (*n* = 12)	Mreg + MSC (*n* = 12)	*p*-Value^b^	Mreg (*n* = 8)	Mreg + MSC-EV (*n* = 8)	*p*-Value^c^
**Arachidonic acid-derived**									
TxB_2_	369	169	195	15.5 (13.8)	18.4 (11.2)	1.000	19.6 (12.8)	12.1 (13.2)	0.547
PGE_2_	351	271	189	0.3 (0.9)	4.2 (2.8)	0.002	0.0 (0.6)	0.6 (0.9)	0.031
PGD_2_	351	189	233	0.0 (0.0)	0.0 (0.2)	0.250	ND	ND	ND
15-Deoxy-Δ^12,14^-PGJ_2_	315	271	203	ND	ND	ND	ND	ND	ND
LTB_4_	335	195	59	ND	ND	ND	ND	ND	ND
LXA_4_	351	115	217	ND	ND	ND	ND	ND	ND
15-HETE	319	219	301	0.5 (1.0)	0.8 (1.0)	0.020	0.6 (0.1)	0.8 (0.8)	0.313

**Eicosapentaenoic acid-derived**									
18-HEPE	317	215	259	0.4 (0.4)	0.4 (0.1)	0.193	0.5 (0.3)	0.4 (0.3)	0.148

**Docosahexaenoic acid-derived**									
RvD1	375	215	233	ND	ND	ND	ND	ND	ND
RvD2	375	215	175	ND	ND	ND	ND	ND	ND
RvD3	375	147	137	ND	ND	ND	ND	ND	ND
10*S*,17*S*-diHDHA	359	153	206	ND	ND	ND	ND	ND	ND
MaR1	359	177	250	ND	ND	ND	ND	ND	ND
17-HDHA	343	245	201	0.7 (1.2)	1.1 (1.6)	0.009	1.0 (0.5)	0.9 (0.7)	0.945
14*S*-HDHA	343	205	161	0.4 (0.4)	0.4 (0.7)	0.359	0.3 (0.2)	0.4 (0.6)	0.078

*diHDHA, dihydroxydocosahexaenoic acid; EV, extracellular vesicle; HDHA, hydroxydocosahexaenoic acid; HEPE, hydroxyeicosapentaenoic acid; HETE, hydroxyeicosatetraenoic acid; IQR, interquartile range; LT, leukotriene; LX, lipoxin; MaR, maresin; Mreg, regulatory macrophage; MSC, mesenchymal stromal cell; ND, not detected; PG, prostaglandin; Qual, qualifier; Quant, quantifier; Rv, resolvin; Tx, thromboxane; w/o, with or without*.

*^a^The LMs were identified using liquid chromatography–tandem mass spectrometry method with detection limit <0.2 ng/ml*.

*^b^The statistical significance of variation between Mreg-conditioned media and Mreg-conditioned media with MSC coculture was determined using the Wilcoxon matched-pairs signed-rank test*.

*^c^The statistical significance of variation between Mreg-conditioned media and Mreg-conditioned media with MSC-derived EVs was determined using the Wilcoxon matched-pairs signed-rank test*.

TxB_2_, PGE_2_, 15-HETE, 18-HEPE, 17-HDHA, and 14*S*-HDHA were also detected both from MSC-conditioned media and MSC-EVs (Figure S7 in Supplementary Material).

### Effect of MSC Coculture on the Phagocytic Ability of Mreg

**Figure 5:**
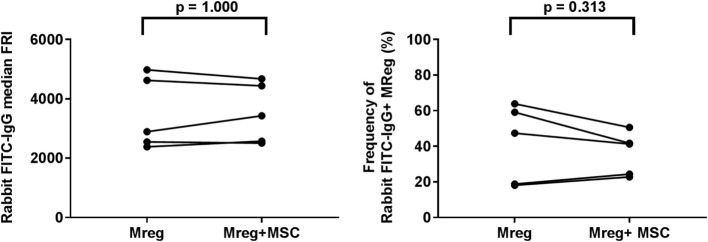
Effect of MSC coculture on the phagocytic ability of Mreg. The phagocytic ability of Mregs was assessed using latex beads coated with FITC-labeled rabbit IgG. The median FRI (left panel) and frequencies of positive cells (right panel) were determined with flow cytometry analysis. The variations between Mreg with and without MSC coculture were analyzed by the Wilcoxon matched-pairs signed-rank test. The number of biological replicates is 5. Abbreviations: FRI, fluorescence intensity; IgG, immunoglobulin G; Mreg, regulatory macrophage; MSC, mesenchymal stromal cell.

The phagocytic ability of Mregs was assessed using latex beads coated with FITC-labeled rabbit IgG. It was noted that mannose receptor CD206 expression of Mregs was highly upregulated by the addition of the immune complex in all experimental conditions, and after 4-h incubation, the median frequencies of CD206^+^ Mregs were >90% (data not shown). However, there were neither significant differences in median FRI of FITC-IgG (*p* = 1.000) nor in frequency of FITC-IgG^+^ Mreg cells (*p* = 0.313) between Mregs culture alone and with MSC coculture (Figure 5).

## Discussion

Active dampening of the inflammatory response is a key phenomenon in the restoration of tissue homeostasis after infection or tissue damage. Resolution-phase macrophages orchestrate anti-inflammatory actions by secreting various factors, including IL-10 and SPMs (7). In this study, we investigated the effects of immunomodulative human bone marrow-derived MSCs and MSC-EVs on human Mregs. We observed that both MSC coculture and MSC-EVs enhanced the anti-inflammatory phenotype of Mreg by downregulating the production of IL-23 and IL-22. Several LMs and pathway markers were identified from Mreg-, MSC-, and EV-conditioned media. Interestingly, our results indicate that MSC-EVs may also mediate the discovered changes in cytokine levels *via* PGE_2_, and thus promote the resolution of inflammation.

Macrophage polarization and activation status strongly depend on the microenvironmental stimuli. Currently, the nomenclature system in the research field is heterogeneous and further confusion may arise from the fact that many murine markers fail to translate to human macrophages (4, 5). In the literature, “regulatory,” “Mreg,” or “immunosuppressive” macrophages comprise populations with a spectrum of characteristics evoked by different experimental conditions (6, 10). Common features include high IL-10 and TGF-β production. Mregs are also potent antigen-presenting cells and express co-stimulatory molecules needed for T and B cell activation (38). In our study, we characterized the phenotype of human Mregs and compared it with those of the conventionally generated M1 and M2 populations. In concordance with the previous Mreg studies, the Mregs generated in our experiments produced substantially higher levels of IL-10 than M1 and M2 populations (10, 11). Despite the activation by M1-stimulus-like combination of IFN-γ and LPS, the phenotype and cytokine profile of Mregs resembled more M2 than M1. The levels of pro-inflammatory cytokines, such as TNF-α and IL-23, were markedly lower in Mreg-conditioned media than in M1-conditioned media, but higher that in M2-conditioned media. As expected, the variance in biological replicates was rather high due to differences in PBMC donors. However, the observed Mreg phenotype CD80^low/intermediate^, CD86^+^, CD163^low^, and CD206^low^ resembled previous reports for the shared markers (6, 13).

It has been reported that MSCs modify the differentiation of monocyte and macrophage populations toward anti-inflammatory or M2 phenotype, by, e.g., increasing IL-10 production, the expression of scavenger receptors, and phagocytic ability (23–26, 39, 40). However, the majority of the work has been done either with mouse models, cell lines, or in conditions where macrophages are not pre-polarized. Therefore, the previous observations are not directly applicable to our findings due to the different phenotype of Mregs. We are the first to show the effect of MSCs and MSC-EVs on fully differentiated human Mregs in the presence of activation medium (LPS and IFN-γ). MSCs have been shown to decrease TNF-α secretion from macrophages (39). In Mreg population, we observed a tendency for decreasing TNF-α. The reduction was much more prominent in the M1 population (data not shown) that produced this cytokine at a very high level. Furthermore, in contrast to the findings in M1/M2 axis, we detected that MSC coculture actually decreased the very high level of IL-10 in the conditioned media.

Both MSC coculture and MSC-EVs reduced the expression of CD163 and CD206 in Mregs, which differs from the previous M1/M2 axis results showing increased expression of M2 markers CD163 and CD206 (25, 39, 40). This observation is in accordance with the finding that MSCs did not enhance the FcγR-mediated IgG-binding phagocytosis of Mregs. Overall, phagocytosis is carried out by both opsonic (i.e., FcγR) and non-opsonic (i.e., CD206) receptors, and the phagocytosis ability, especially of Mregs, could be reasonably investigated using apoptotic neutrophils, which are highly abundant at the resolution phase (7). Altogether, the differences in the MSC-derived effects reflect the nature of diverse macrophage populations, the variation in species, and other experimental conditions. Most likely, the role of Mreg *in vivo* is not primarily conventional phagocytosis, but more related to efferocytosis and immune regulation.

Our novel results indicate that both MSC coculture and MSC-EVs are able to induce reduction in the levels of IL-23 and IL-22 and thereby enhance the anti-inflammatory characteristics of Mregs. IL-23 is a pro-inflammatory cytokine that induces and maintains the Th17 effector T cell population and pathogenicity, and many chronic immune-mediated disorders, such as psoriasis, arthritis, and Crohn’s disease, are strongly associated with IL-23 dysregulation (41–44). On the other hand, IL-22 is a major secretory product of Th17, and the function of IL-22 has been reported to mediate IL-23-induced inflammation and crosstalk between tissue barrier cells and the cells of the immune system (45). Thus, reduction of both IL-23 and IL-22 could decrease the induction of highly pathogenic Th17 cells and thus exacerbation of inflammation (46). Intriguingly, it has been recently reported that Th17 cells contribute to the resolution of inflammation by differentiating into T regulatory type 1 cells (47). We can therefore hypothesize that MSC coculture and MSC-EVs treated Mreg populations may promote resolution *via* reduction of Th17 pathogenicity or Th17 conversion. The hypothesis is supported by findings by Chiossone et al. (25), who reported induction of CD25^high^Foxp3^+^ regulatory T cells by MSC-educated macrophages and by Melief et al. (48) presenting that induction of regulatory T cells by MSC involves skewing monocytes toward M2-type macrophages.

Specialized proresolving mediators are synthetized from n-6 and n-3 fatty acids such as arachidonic acid, eicosapentaenoic acid, and docosahexaenoic acid. The biosynthetic pathways of SPMs include multiple intermediates, which may also serve as pathway markers (49). We identified TxB_2_, PGE_2_, 15-HETE, 18-HEPE, 17-HDHA, and 14*S*-HDHA from the Mreg-conditioned media with both MSC coculture and MSC-EVs. These molecules belong to the reported LM profiles produced by human macrophages (16). We could not detect any proresolving end products, the levels of which likely remained under the detection limit of our LC–MS/MS system. Although a well-known macrophage-derived SPM, maresin 1, was not detected with the current method, a maresin pathway marker, 14*S*-HDHA, was identified. Generally, monohydroxy pathway markers are more stable than SPM end products.

We observed that MSCs and, for the first time, MSC-EVs increased the PGE_2_ production in the Mreg coculture. Conventionally, the PGE_2_ has been regarded as a pro-inflammatory molecule participating in the initiation of inflammation. However, PGE_2_ also possesses immunosuppressive properties (50, 51) and is considered one of the most potent immunosuppressive mechanisms of MSCs (21), shown also in the context of macrophage polarization by MSCs (23–25). The dual function of PGE_2_ might arise from the differing activities of PGE_2_ receptors (EPs). Poloso et al. recently reported that PGE_2_ regulates the IL-23 release in human monocyte-derived dendritic cells and that at low PGE_2_ concentrations the high-affinity EP4 increased IL-23 production (52). Another receptor, EP2 has been suggested to respond to high PGE_2_ amounts by downregulating the IL-23 release (52, 53). The mechanism *via* the EP2 may partly explain the observations of high PGE_2_ and decreased IL-23 in our study. The fact that MSC-EVs are also able to induce the production of PGE_2_ from responder cells represents an interesting novel mechanism of action supported by the findings of Liu et al. (54).

Prostaglandin E_2_ has been shown to induce LM class switching in neutrophils. Modulation of 5- and 15-LOX expressions leads to the inhibition of pro-inflammatory leukotriene B_4_ and increased production of proresolving LXA_4_ and its pathway marker 15*S*-HETE in neutrophils (14). In this study, the level of 15-HETE was increased in the coculture of Mreg and MSC, which may imply that the elevated concentration of PGE_2_ acted as a cue for LM class switching. During the course of resolution, other SPMs, such as Rvs, protectins, and maresins, are produced (15). Interestingly, RvD1, RvD2, and RvE1 have been reported to reduce IL-23 production in asthma, microbial peritonitis, and allergic airway mouse models, respectively (55–57). These findings suggest a possible association between the PGE_2_-induced class switching and the observed reduced IL-23 production.

Human Mregs have been investigated in cell-based immunosuppressive therapy in early-phase clinical trials in renal transplantations (6, 13, 58), and altogether, the need for the development of cell-based medicinal products is increasing. Modification of Mregs into enhanced tolerance-promoting phenotype is of interest, and our results displaying the reduction of IL-23 and IL-22, support a potential role for combining therapeutic Mregs with MSC or MSC-EVs. The underlying mechanisms, including the MSC-EV-derived induction of PGE_2_ production and the possible conversion of Th17 into regulatory T cells, require further studies.

## Conclusion

In this study, we demonstrate that human MSCs and MSC-EVs are capable of inducing proresolving changes in mature human Mregs. Both MSCs and MSC-EVs decrease the production of pro-inflammatory IL-23 and IL-22 while increasing immunosuppressive PGE_2_ production. Our findings suggest that MSCs and MSC-EVs may potentiate the proresolving phenotype of Mregs and supports the rationale of further studying the underlying mechanisms. Priming of Mregs with MSC or MSC-EVs is a potential novel approach for promoting the efficacy of Mreg therapy.

## Ethics Statement

BM MSC donors gave their voluntary, informed, and written consent before sample collection, and the study protocols were approved by the Ethical Committee of Northern Ostrobothnia Hospital District or Ethical Committee of Hospital District of Helsinki and Uusimaa, Finland. The utilization of anonymized PBMNCs from blood donors in research is in accordance with the rules of the Finnish Supervisory Authority for Welfare and Health (Valvira).

## Author Contributions

KH, MH, SL, and EK designed the study. PL and MK provided human MSCs for the study. KH, VS, and MH completed the laboratory analyses. HR, MH, and RK set up the LC–MS/MS method. MH completed the lipid mediator analyses. KH performed the most of the data analyses. KH and MH interpreted the results and wrote the manuscript. SL, HR, RK, PL, MK, and EK critically revised the manuscript and contributed to discussion. All the authors approved the final manuscript.

## Conflict of Interest Statement

The authors declare that the research was conducted in the absence of any commercial or financial relationships that could be construed as a potential conflict of interest.
