# Supplementary material for: Mesenchymal Stromal Cells and Their Extracellular Vesicles Enhance the Anti-Inflammatory Phenotype of Regulatory Macrophages by Downregulating the Production of Interleukin (IL)-23 and IL-22
Source: Front Immunol. 2018 Apr 12;9:771. doi: 10.3389/fimmu.2018.00771 (PMC5906545; doi:10.3389/fimmu.2018.00771)
Supplement: Supplementary file 1 [file Data_Sheet_1.docx]

Supplementary Material

Mesenchymal stromal cells and their extracellular vesicles enhance the anti-inflammatory phenotype of regulatory macrophages by down-regulating the production of IL-23 and IL-22

**Kati Hyvärinen*, Minna Holopainen, Vita Skirdenko, Hanna Ruhanen, Petri Lehenkari, Matti Korhonen, Reijo Käkelä, Saara Laitinen, Erja Kerkelä**

***Correspondence:** Kati Hyvärinen: [kati.hyvarinen@bloodservice.fi](mailto:kati.hyvarinen@bloodservice.fi)

# Supplementary Methods

**Macrophage polarization assay**

*Isolation of peripheral blood mononuclear cells, day 0*

Peripheral blood mononuclear cells (PBMC) were extracted from buffy coats using density gradient centrifugation. The extraction was executed using SepMate™ tubes (STEMCELL Technologies) according to the manufacturer’s protocol or using conventional method. Buffy coat sample was diluted 1:2 with endotoxin-free PBS w/o Ca^2+^/Mg^2+^ and 30 ml of suspension was carefully layered over 15 ml of Ficoll-Pague^TM^ Plus (GE Healthcare Life Sciences) in 50 ml tubes. The samples were centrifuged at 400 g for 40 min at room temperature (RT) without brake. After centrifugation, the mononuclear cell layer was transferred to new tube, suspended into 45 ml PBS, and centrifuged at 200 g for 10 min for removal of platelets. The acquired pellet was washed with 45 ml PBS followed by centrifugation at 350 g for 10 min twice. Finally, the cells were suspended with 5 ml PBS, 30 µm filtered, and counted with NucleoCounter®.

*Monocyte selection and polarization, days 0 to 6*

Monocyte selection was performed by plating 2 x 10^6^ PBMC/well in Nuclon Delta Surface 24 well plates (Thermo Scientific) in 1 ml RPMI Medium 1640 (Gibco), incubating at 37 °C, 5% CO_2_ for 2 h, and washing the cells with 1 ml warm endotoxin-free PBS w/o Ca^2+^/Mg^2+^ twice. After washing, the attached monocytes were incubated at 37 °C, 5% CO_2_ for six days in the following Polarization Media: 1 ml 5 ng/ml M-CSF (PromoCell), 10% FBS (Sigma-Aldrich) in RPMI Medium 1640, GlutaMAX^TM^ Supplement for Mreg polarization; 1 ml 50 ng/ml GM-CSF (PromoCell), 10% FBS in RPMI Medium 1640, GlutaMAX^TM^ Supplement for M1 polarization; and 1 ml 50 ng/ml M-CSF, 10% FBS in RPMI Medium 1640, GlutaMAX^TM^ Supplement for M2 polarization.

At day six, the media were replaced with the following Activation Media: 25 ng/ml IFN-γ, 10 ng/ml LPS, 5 ng/ml M-CSF, 10% FBS in RPMI Medium 1640, GlutaMAX^TM^ Supplement for Mreg polarization; 50 ng/ml IFN-γ, 10 ng/ml LPS, 50 ng/ml GM-CSF, 10% FBS in RPMI Medium 1640, GlutaMAX^TM^ Supplement for M1 polarization; and 20 ng/ml IL-4, 50 ng/ml M-CSF, 10% FBS in RPMI Medium 1640, GlutaMAX^TM^ Supplement for M2 polarization. The added media volume was 500 µl in treatment wells and 600 µl in control wells. The incubation was continued at 37 °C, 5% CO_2_.

*MSC co-culture or MSC-EV supplementations, days 7 to 10*

At day seven, 20 000 MSCs (at passage six) in 100 µl Mreg/M1/M2 Activation Media were added into representative treatment wells. The final media volume was 600 µl/well, and the cells were incubated at 37 °C, 5% CO_2_ for three days.

Alternatively, in MSC-EV supplementation experiments, at day seven, isolated EVs were suspended in 1600 µl Mreg Activation Media and 50 µl were added into 30 representative treatment wells. The supplementation was repeated at day nine, isolated EVs in 50 µl representative Mreg Activation Media were added into each well. The final media volume was 600 µl/well and the cells were incubated at 37 °C, 5% CO_2_ for three days.

*Media and cell sample collection, day 10*

Media samples were collected from each well and centrifuged at 300 g for 15 min at RT. The supernatants were snap frozen using dry ice and stored at -70 °C. Attached cells were washed with 1 ml warm PBS twice. Cell samples for flow cytometry analysis were detached with 0.5 ml/well cold Macrophage Detachment Solution DFX (PromoCell) by pipetting gently and monitoring the detachment process with microscope. The cells were suspended up to 15 ml PBS, and centrifuged at 350 g for 10 min. After centrifugation, the cells were washed with 2 ml 0.3% bovine serum albumin, 2 mM EDTA in PBS, pH 7.2 followed by centrifugation at 350 g for 10 min.

# Supplementary Figures and Tables

## Supplementary Table 1. Retention times, multiple reaction monitoring conditions, and internal standard for lipid mediators in the LC-MS/MS method.

| **Lipid Mediator** | | **Retention time (min)** | **Q1 (Precursor Ion)** | **Q3 (Quantifier)** | **CE (V)** | **Q3 (Qualifier)** | **CE (V)** | **Internal standard** |
| --- | --- | --- | --- | --- | --- | --- | --- | --- |
| Arachidonic acid-derived | |  |  |  |  |  |  |  |
|  | TxB_2_ | 2.34 | 369 | 169 | 12 | 195 | 10 | d_4_-PGE_2_ |
|  | PGE_2_ | 2.75 | 351 | 271 | 16 | 189 | 18 | d_4_-PGE_2_ |
|  | d_4_-PGE_2_ | 2.76 | 355 | 275 | 14 | 193 | 16 |  |
|  | PGD_2_ | 2.93 | 351 | 189 | 18 | 233 | 8 | d_4_-PGE_2_ |
|  | 15-deoxy-Δ^12,14^-PGJ_2_ | 5.50 | 315 | 271 | 10 | 203 | 18 | d_4_-PGE_2_ |
|  | LTB_4_ | 4.37 | 335 | 195 | 12 | 59 | 16 | d_4_-PGE_2_ |
|  | LXA_4_ | 3.22 | 351 | 115 | 12 | 217 | 16 | d_5_-RvD2 |
|  | 15-HETE | 6.07 | 319 | 219 | 10 | 301 | 8 | d_4_-PGE_2_ |
| Eicosapentaenoic acid-derived | |  |  |  |  |  |  |  |
|  | 18-HEPE | 5.37 | 317 | 215 | 10 | 259 | 8 | d_4_-PGE_2_ |
| Docosahexaenoic acid-derived | |  |  |  |  |  |  |  |
|  | RvD1 | 3.27 | 375 | 215 | 16 | 233 | 10 | d_5_-RvD2 |
|  | RvD2 | 3.00 | 375 | 215 | 14 | 175 | 16 | d_5_-RvD2 |
|  | d_5_-RvD2 | 2.99 | 380 | 175 | 22 | 277 | 10 |  |
|  | RvD3 | 2.88 | 375 | 147 | 16 | 137 | 20 | d_5_-RvD2 |
|  | 10*S*,17*S*-diHDHA | 4.33 | 359 | 153 | 12 | 206 | 12 | d_5_-RvD2 |
|  | MaR1 | 4.33 | 359 | 177 | 14 | 250 | 10 | d_5_-RvD2 |
|  | 17-HDHA | 6.14 | 343 | 245 | 8 | 201 | 12 | d_4_-PGE_2_ |
|  | 14*S*-HDHA | 6.25 | 343 | 205 | 8 | 161 | 10 | d_4_-PGE_2_ |

CE, collision energy; diHDHA, dihydroxydocosahexaenoic acid; HDHA, hydroxydocosahexaenoic acid; HEPE, hydroxyeicosapentaenoic acid; HETE, hydroxyeicosatetraenoic acid; LC-MS/MS, liquid chromatography-tandem mass spectrometry; LT, leukotriene; LX, lipoxin; MaR, maresin; PG, prostaglandin; Rv, resolvin; Tx, thromboxane.

## Supplementary Figures

**Mreg w/o MSC-EVs**

PBMC extraction, day 0

Monocyte selection, day 0

Polarization Media, day 0

Activation Media, day 6

EV supplementation, day 7

EV supplementation, day 9

Sample collection and flow cytometry analysis, day 10

**Mreg w/o MSC co-culture**

PBMC extraction, day 0

Monocyte selection, day 0

Polarization Media, day 0

Activation Media, day 6

MSC co-culture, day 7

Sample collection and flow cytometry analysis, day 10

**Supplementary Figure 1.** **Schematic overview of macrophage polarization assay.** EV, extracellular vesicle; Mreg, regulatory macrophage; MSC, mesenchymal stromal cell; PBMC, peripheral blood mononuclear cells; w/o, with or without.


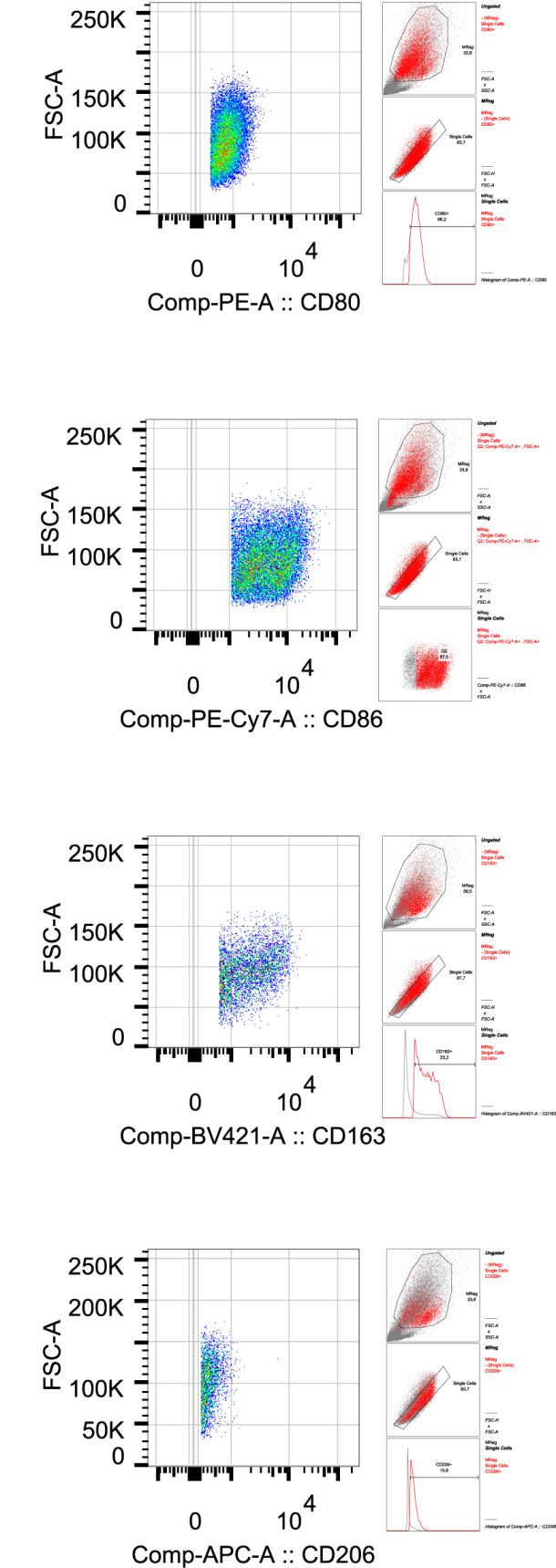


**Supplementary Figure 2. Representative gating strategy in flow cytometry analysis.** Representative backgating figures for Mregs with fluorescent label-conjugated antibodies depicting the gating strategy. The antibody staining was performed with PE-CD80, PE-Cy7-CD86, BV421-CD163, and APC-CD206 according to the manufacturers’ instructions.


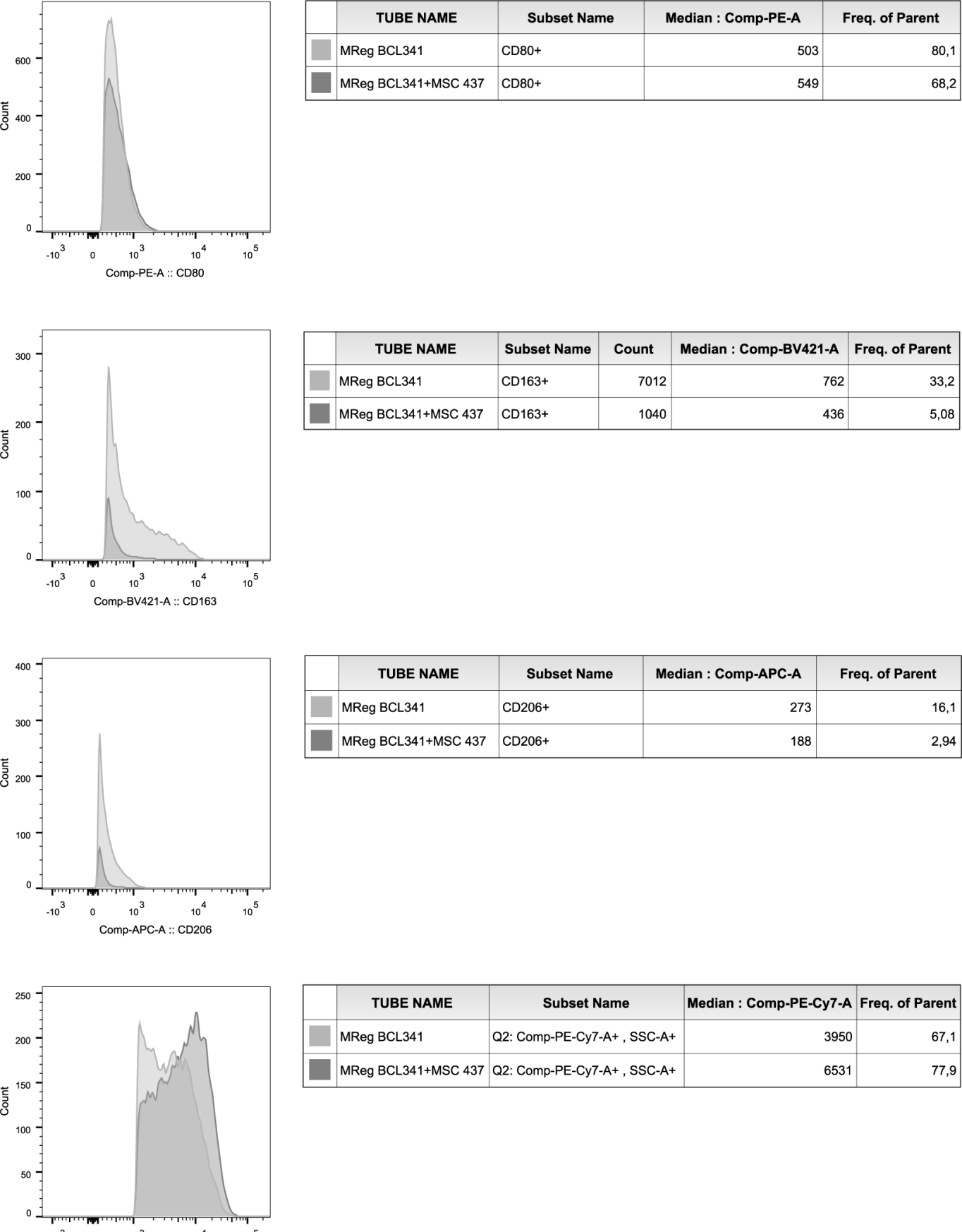


**Supplementary Figure 3. Mreg w/o MSC co-culture raw data overlays.** The antibody staining was performed with PE-CD80, PE-Cy7-CD86, BV421-CD163, and APC-CD206 according to the manufacturers’ instructions. The median fluorescence intensities and frequencies of positive cells were determined with flow cytometry analysis.

**
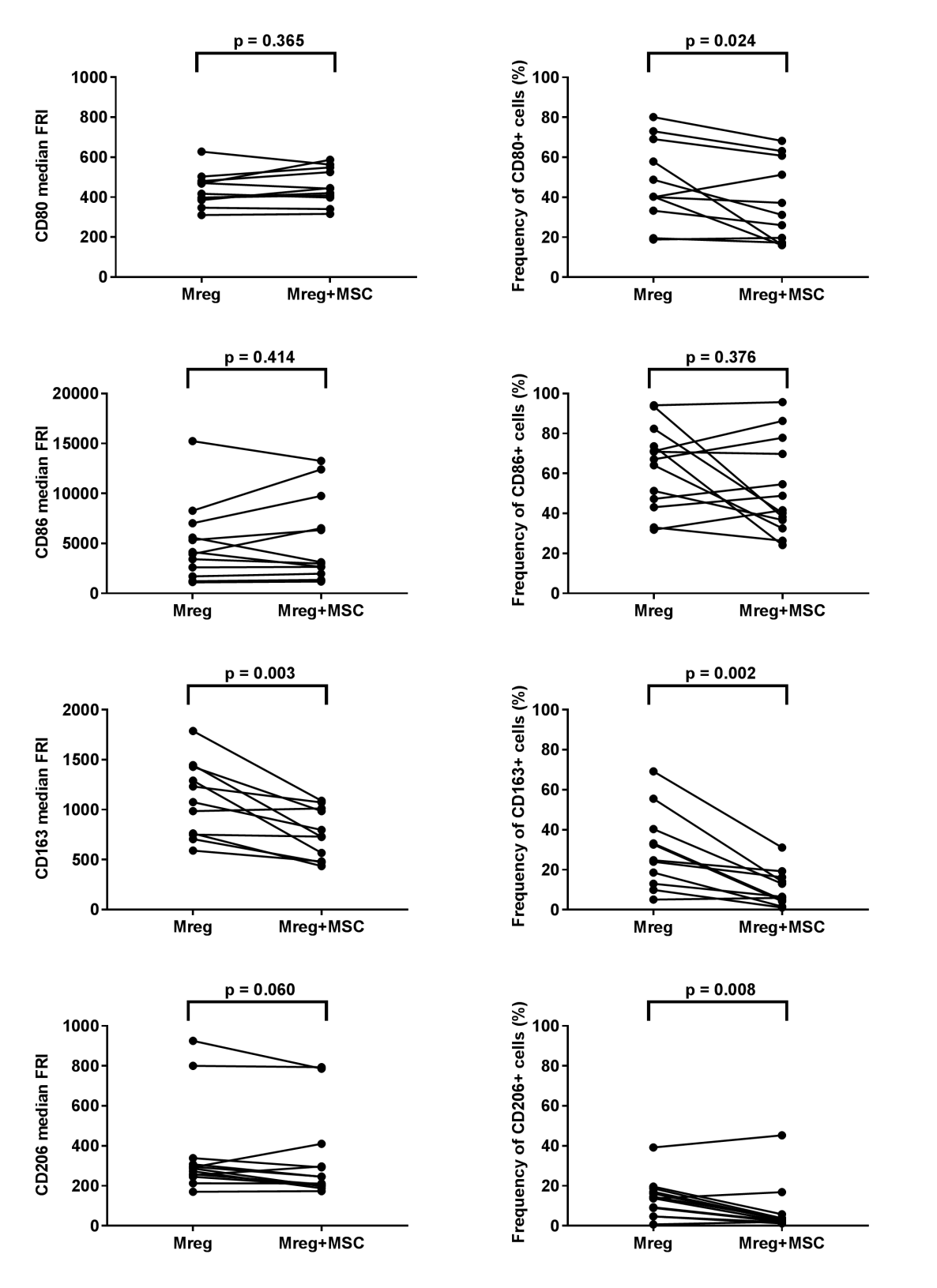
**

**Supplementary Figure 4. Effect of MSC co-culture on Mreg phenotype.** The antibody staining was performed with PE-CD80, PE-Cy7-CD86, BV421-CD163, and APC-CD206 according to the manufacturers’ instructions. The median fluorescence intensities (left panel) and frequencies of positive cells (right panel) were determined with flow cytometry analysis. The significance of variation between Mreg with and without MSC co-culture was analyzed using the Wilcoxon matched-pairs signed-rank test. The number of biological replicates varied from 11 to 13. FRI, fluorescence intensity; Mreg, regulatory macrophage; MSC, mesenchymal stromal cell.


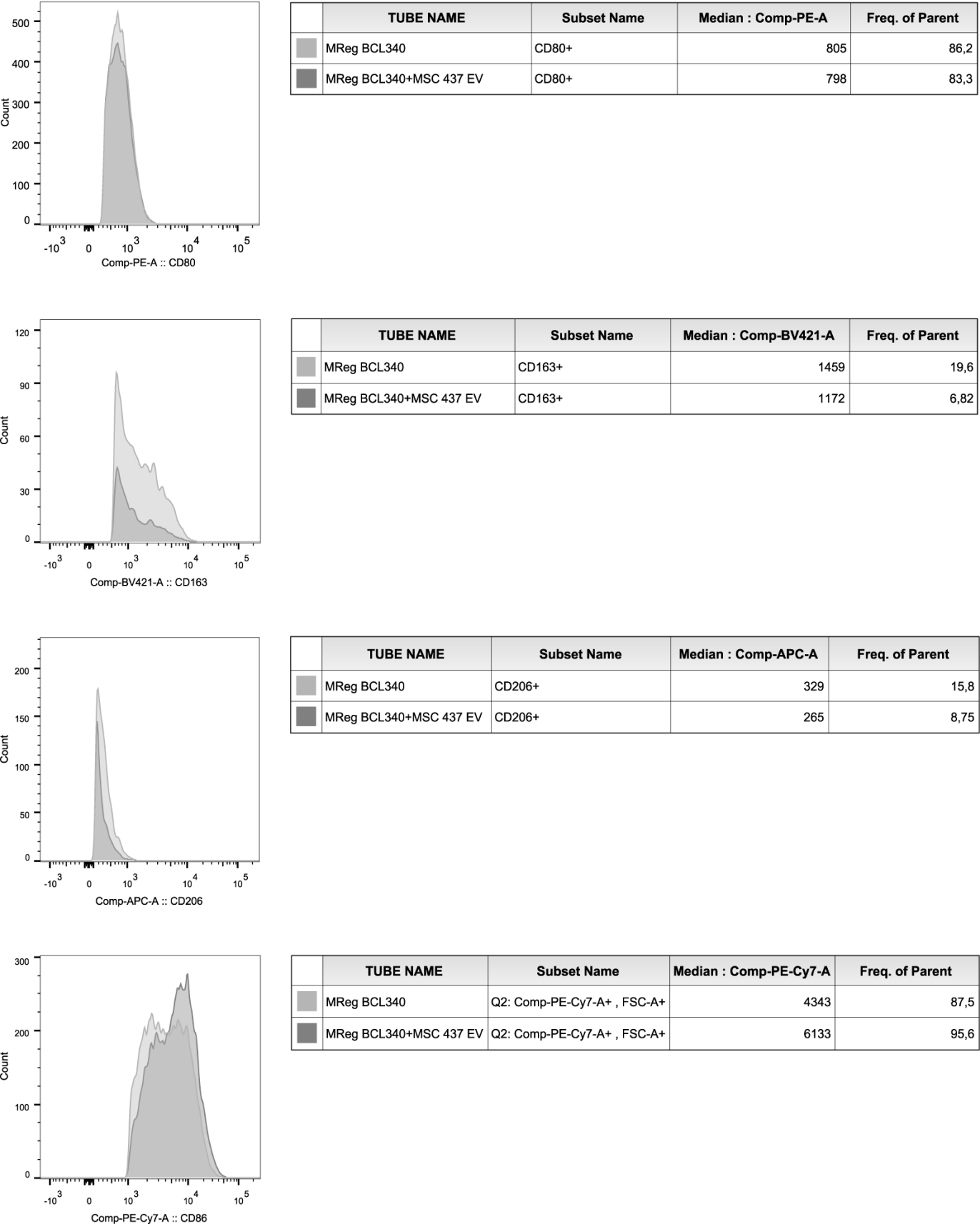


**Supplementary Figure 5. Mreg w/o MSC-EVs raw data overlays.** The antibody staining was performed with PE-CD80, PE-Cy7-CD86, BV421-CD163, and APC-CD206 according to the manufacturers’ instructions. The median fluorescence intensities and frequencies of positive cells were determined with flow cytometry analysis.

**
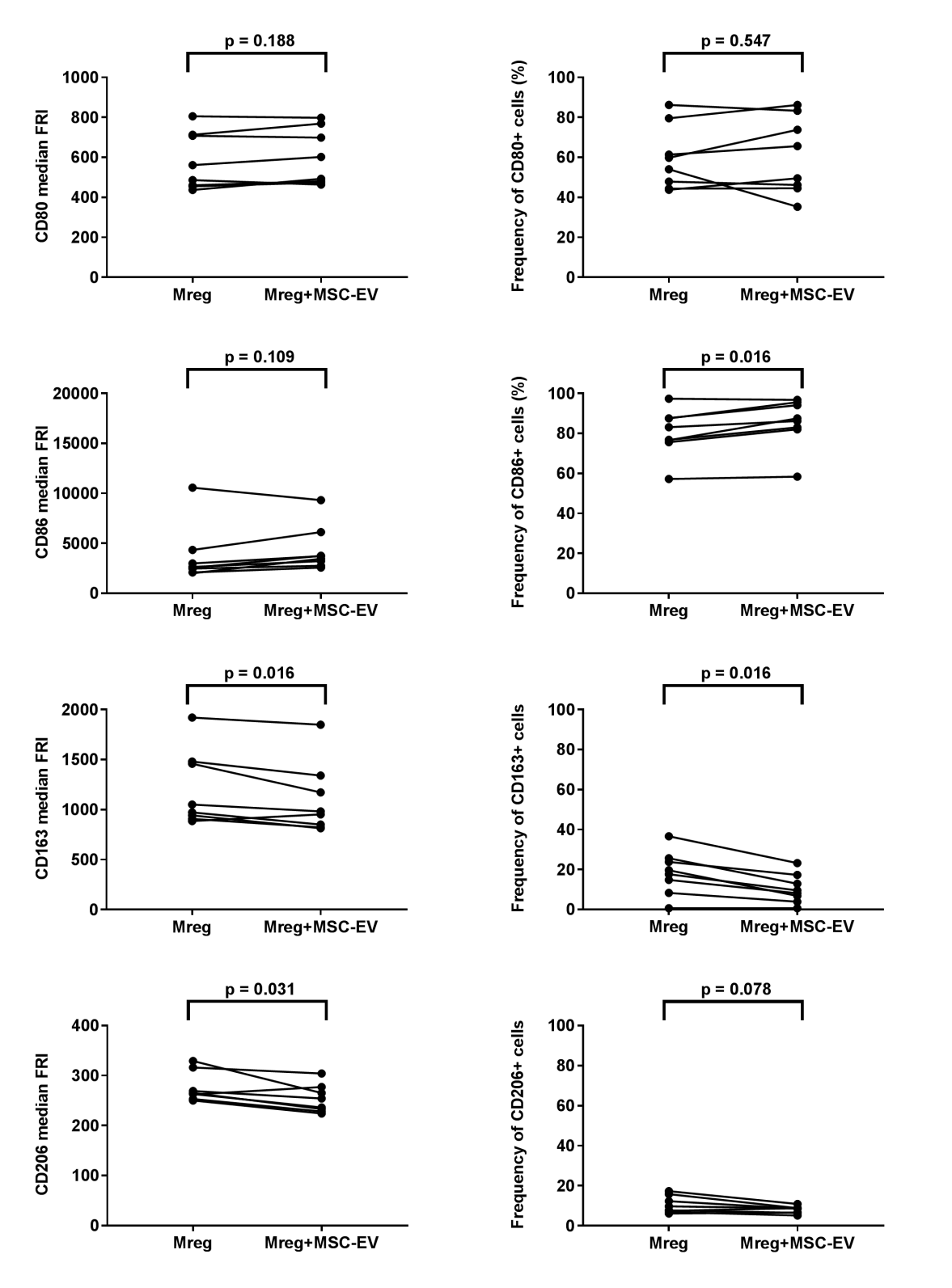
**

**Supplementary Figure 6. Effect of MSC-EVs on Mreg phenotype.** The antibody staining was performed with PE-CD80, PE-Cy7-CD86, BV421-CD163, and APC-CD206 according to the manufacturers’ instructions. The median fluorescence intensities (left panel) and frequencies of positive cells (right panel) were determined with flow cytometry analysis. The significance of variation between Mreg with and without MSC-derived extracellular vesicles was analyzed using the Wilcoxon matched-pairs signed-rank test. The number of biological replicates was eight. EV, extracellular vesicle; FRI, fluorescence intensity; Mreg, regulatory macrophage; MSC, mesenchymal stromal cell.


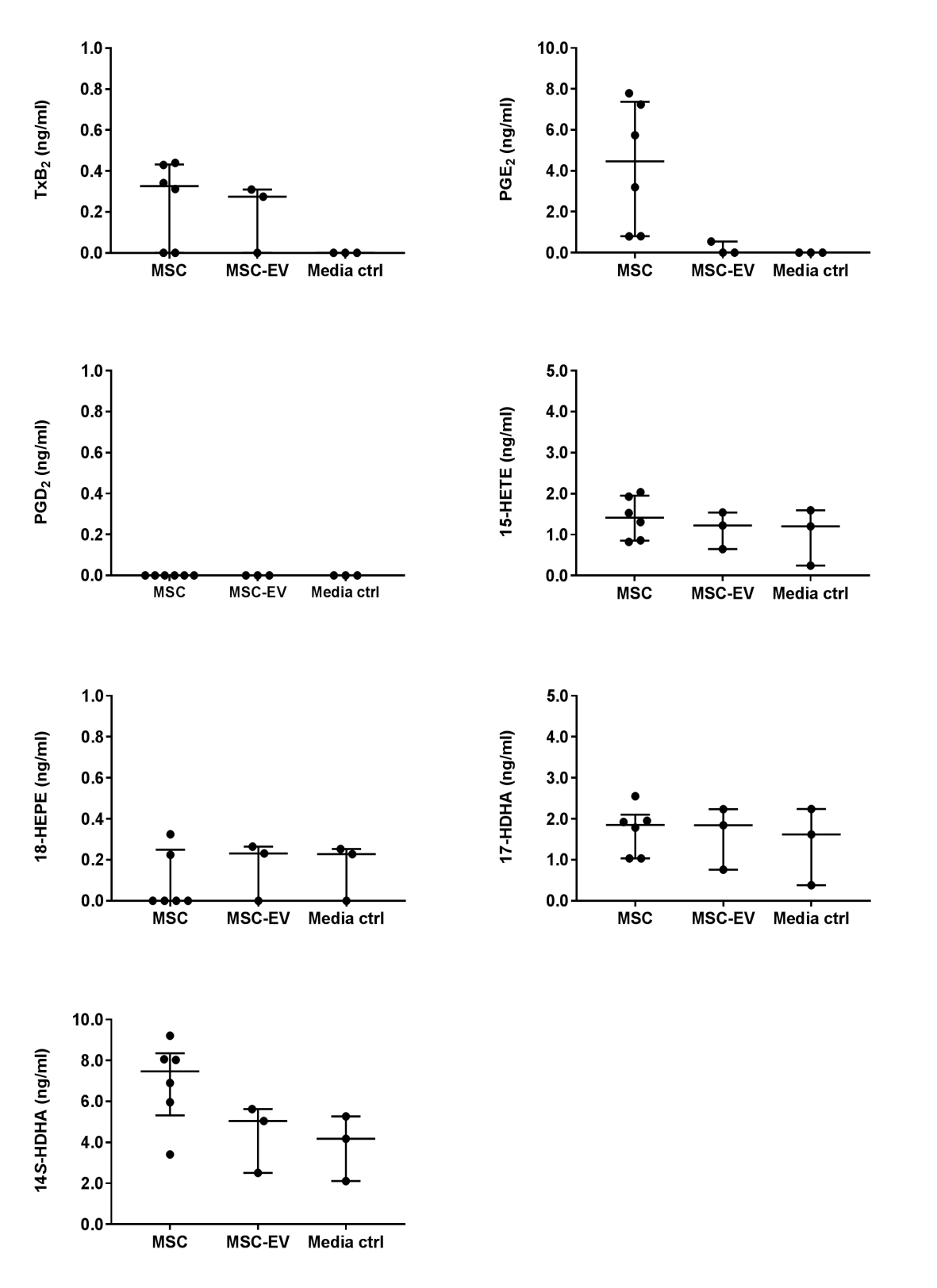


**Supplementary Figure 7. Identified lipid mediators in MSC-conditioned media and MSC-EVs.** The levels of lipid mediators were determined using liquid chromatography-tandem mass spectrometry method. Mreg Activation Media: 25 ng/ml IFN-γ, 10 ng/ml LPS, 5 ng/ml M-CSF, 10% FBS in RPMI Medium 1640, GlutaMAX^TM^ Supplement. The number of biological replicates varies from 3 to 6. The results are presented as median with interquartile range. Ctrl, control; EV, extracellular vesicle; HDHA, hydroxydocosahexaenoic acid; HEPE, hydroxyeicosapentaenoic acid; HETE, hydroxyeicosatetraenoic acid; MSC, mesenchymal stromal cell; PG, prostaglandin; Tx, thromboxane.
